# Takotsubo Cardiomyopathy (TCM) After Uncomplicated Paraesophageal Hernia Repair: A Case Report and Review on Postoperative TCM

**DOI:** 10.7759/cureus.41770

**Published:** 2023-07-12

**Authors:** Chin Fung Kelvin Kan, Bianca Rich, Noah Brown, Sophia Janes, Joanna Grudziak

**Affiliations:** 1 Anesthesiology, University of Utah School of Medicine, Salt Lake City, USA; 2 General Surgery, University of Utah School of Medicine, Salt Lake City, USA; 3 Obstetrics and Gynecology, University of Utah School of Medicine, Salt Lake City, USA

**Keywords:** postoperative takotsubo cardiomyopathy, perioperative takotsubo cardiomyopathy, toupet fundoplication, paraesophageal hernia repair, takotsubo cardiomyopathy

## Abstract

Takotsubo cardiomyopathy (TCM) is a rare stress-induced condition that appears rarely in suspected acute myocardial infarction cases. It causes unexplained left ventricular failure, but most cases are reversible with supportive treatment. In this report, we present the case of a 70-year-old female who developed acute hypotension after a laparoscopic Toupet fundoplication on postoperative day one, requiring care in the surgical intensive care unit. Following consultation with the cardiology service and further imaging and tests, she was diagnosed with TCM. This report outlines the potential mechanisms and management of TCM in the intensive care unit, emphasizing the importance of prompt diagnosis and treatment.

## Introduction

Takotsubo cardiomyopathy (TCM) is a rare form of cardiomyopathy that occurs in 1% to 2% of patients suspected of having acute coronary syndrome (ACS) [1]. It was first identified in Japan in the 1990s and is characterized by acute electrocardiography (EKG) changes, with the left ventriculogram showing hyperkinesis in the basal segments but hypokinesis in the apical, diaphragmatic, and/or anterolateral segments [1]. Takotsubo cardiomyopathy is a diagnosis of exclusion, and cardiac catheterization usually demonstrates unremarkable stenosis in the coronary arteries [1]. Previously documented stressors that can induce TCM include extreme emotions, medications, seizures, and surgeries [2-5]. Here, we present the first case of TCM that developed on postoperative day (POD) 1 after an uncomplicated Toupet fundoplication in a patient with gastroesophageal reflux disease with failed conservative treatment and lifestyle changes. Furthermore, we will review the current diagnostic criteria, treatment options, and risk factors associated with TCM.

## Case presentation

A 70-year-old female with sinus node dysfunction status, post-dual-chamber pacemakers, gastroesophageal reflux disease, and failed lifestyle changes and conservative treatment presented for Toupet fundoplication. Her other significant medical history includes hypertension, hyperlipidemia, and chronic obstructive pulmonary disease, for which she was on 2L of home oxygen via nasal cannula. Her course of anesthesia and surgery was unremarkable. Her transthoracic echocardiography (TTE) completed eight months ago, showed normal left ventricular systolic function with an ejection fraction of 57%. However, the entire anterior septum and mid and apical inferior septum were hypokinetic. Her anesthesia was maintained with sevoflurane. During the operation, she received boluses of 40 mg of ephedrine, 800 mcg of phenylephrine, and 4 units of vasopressin to maintain a mean arterial pressure greater than 65 mmHg. Her post-anesthesia care unit (PACU) stay was unremarkable, and after three hours, she completed PACU phase one and was deemed stable enough to be transferred to the floor.

**Figure 1:**
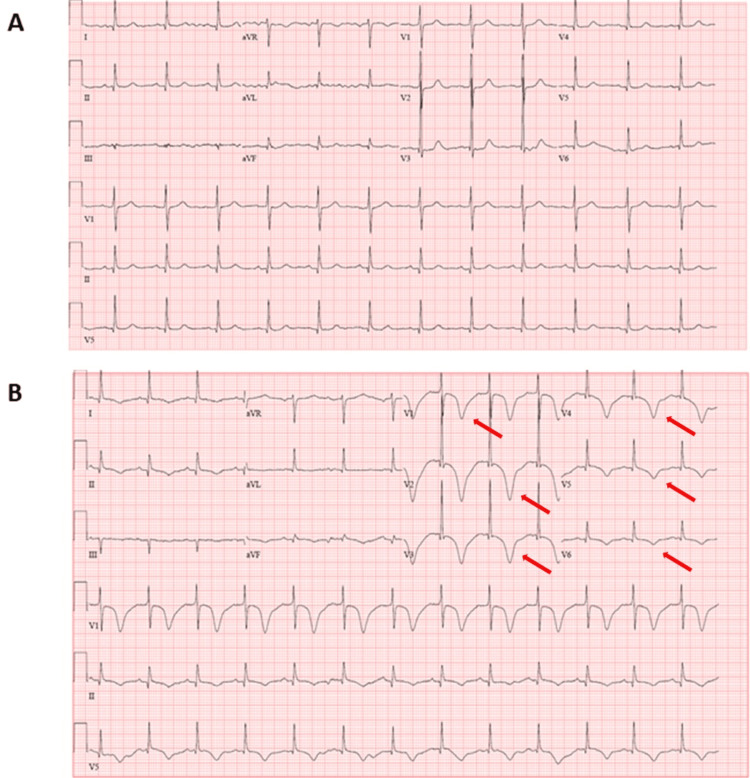
Patient’s EKG at baseline and after developing TCM A: The patient’s baseline EKG shows normal sinus rhythm, which was obtained a month before her surgery; B: The patient’s EKG after she developed TCM shows diffused T-wave inversion (red arrows) and possible anterolateral infarct and anterolateral ischemia EKG: Electrocardiography, TCM: Takotsubo cardiomyopathy

On POD 1, she developed acute severe hypotension and was unresponsive to a crystalloid bolus. Her other vital signs were stable and within normal limits. However, she did endorse middle to low substernal chest pain that was initially localized but later started radiating to her left shoulder. She denied any dizziness, headache, and difficulty breathing. Her physical examination was unremarkable, with weak but palpable radial and dorsalis pedis pulses bilaterally. Her lungs were clear to auscultate, and her abdomen was soft and non-tender. There was no lower extremity edema. Her EKG showed possible anterolateral infarct and anterolateral ischemia (Figure 1). Due to her severe hypotension, she was transferred to the surgical intensive care unit (SICU) for further workup and monitoring.

**Figure 2:**
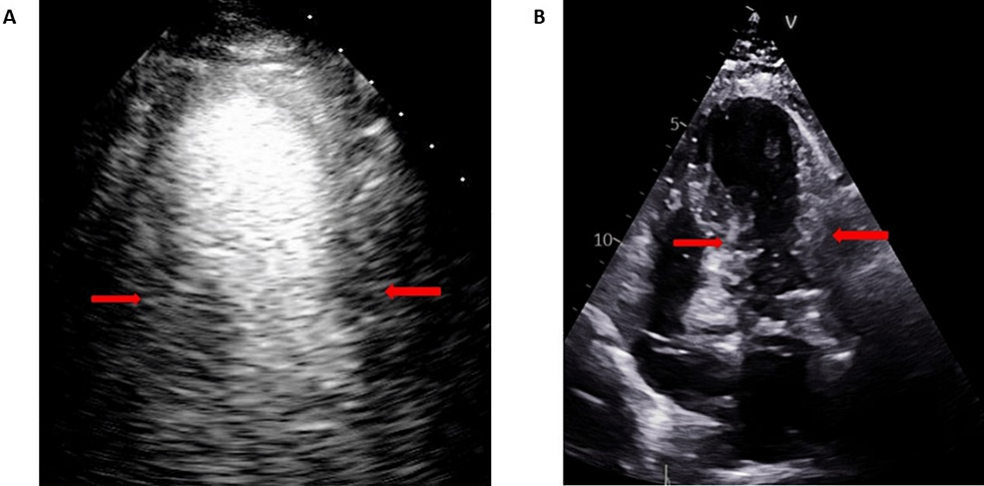
Patient's TTE A: TTE with contrast showing ballooning of left ventricle apex due to severe hypokinesis, left ventricle mid to apical segments with relative hyperkinesis of the base; B) TTE without contrast demonstrated no left ventricle thrombus TTE: Transthoracic echocardiography

Upon admission to the SICU, her troponin level was elevated to 0.35 ng/ml with stable electrolyte levels and hemoglobin. Her BNP increased to 580 pg/ml from a baseline of 34 pg/ml. The TTE showed mildly decreased left ventricular systolic function, with an ejection fraction of 47%. There was severe hypokinesis of the left ventricle mid to apical segments with relative hyperkinesis of the base, which is consistent with TCM (Figure 2).

**Figure 3:**
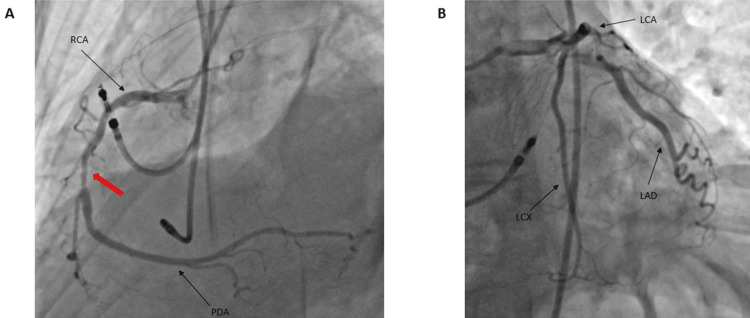
Cardiac catheterization of the patient A: Cardiac catheterization showed 50% to 60% stenosis of the RCA mid-segment but with good blood flow, PDA is unremarkable; B) Cardiac catheterization showed the LCA, LCX, and LAD have no stenosis and are unremarkable RCA: Right coronary artery, PDA: Posterior descending artery, LCA: Left coronary artery, LCX: Left circumflex artery, LAD: Left anterior descending artery

The patient had normal right ventricular size and systolic function. No thrombus was visualized within the left ventricle. Cardiac catheterization showed that her left main, left anterior, and left circumflex coronary arteries had no stenosis, but her right coronary artery had a 50% to 60% stenosis in the mid-segment (Figure 3).

Because her imaging and clinical presentation were consistent with the Mayo criteria, TCM was diagnosed. An arterial line was placed to allow accurate blood pressure measurement and a right internal jugular central line was inserted for long-term pressor infusion and to maintain a mean arterial pressure (MAP) greater than 65 mmHg.

On POD 2, the patient's troponin levels increased to 0.42 ng/ml and she required up to 0.09 mcg/kg/min of epinephrine to meet her MAP goals. By POD 3, the patient's condition had improved, and her epinephrine requirement to maintain the MAP goal began to decrease. Then, on POD 4, she no longer required epinephrine to maintain MAP goals. A new TTE was conducted, which showed that her global left ventricular systolic function remained below normal, but her ejection fraction had improved to 55%. However, her cardiac apex continued to be akinetic, with the apical septal segment and apical lateral segment severely hypokinetic. Additionally, the mid and apical inferior walls, mid anteroseptal segment, mid inferoseptal segment, and mid anterolateral segment were hypokinetic.

On POD 5, the patient was deemed hemodynamically stable, and she was transferred from the SICU to the medicine wards. Her troponin levels had decreased to 0.03 ng/ml by POD 6, and she was started on metoprolol 12.5 mg twice per day. Finally, she was discharged from the hospital on POD 7.

## Discussion

Takotsubo cardiomyopathy, a relatively newly classified cardiomyopathy, was first described in Japan in the 1990s by Sato et al. and Dote et al. [6,7]. Overall, TCM has been reported in approximately 1% to 2% of all patients who present with symptoms of ACS and elevated troponin, and up to 6% of all women presenting with suspected ST-segment elevated myocardial ischemia who undergo urgent angiography [1,8]. There were no clear TCM diagnostic criteria until the publication of the Mayo Criteria in 2004, which was modified in 2008 (Table 1) [9].

**Table 1:** The Mayo criteria for TCM All four criteria must be met for TCM diagnosis [9]. TCM: Takotsubo cardiomyopathy, EKG: Electrocardiography

	Mayo criteria (requires all four criteria)
1	Transient akinesia, hypokinesis, dyskinesis of the left ventricle mid segments with or without apical involvement
2	Absence of obstructive coronary arteries, or angiography evidence of plaque rupture
3	New EKG abnormalities (ST-segment elevation or T-wave inversion) or modest elevation of troponin
4	Absence of pheochromocytoma and myocarditis

Since then, multiple diagnostic criteria have been launched in different countries, including the widely used Heart Failure Association of the European Society of Cardiology (ESC) criteria (Table 2) [10]. In 2018, the International Takotsubo Diagnostic Criteria (InterTAK Diagnostic Criteria) was launched to bring consensus on TCM's diagnostic criteria internationally (Table 3) [8].

**Table 2:** Criteria for TCM laid out by the Heart Failure Association of ESC All seven criteria should be met for TCM diagnosis [10]. ESC: European Society of Cardiology, TCM: Takotsubo cardiomyopathy, LBBB: Left bundle branch block, BNP: Brain natriuretic peptide, NT: N-terminal, LV: Left ventricle, EKG: Electrocardiography

	Heart Failure Association of ESC criteria (requires all seven criteria)
1	Transient regional wall motion abnormalities of left ventricle or right ventricle myocardium, which are frequently, but not always, preceded by a stressful trigger (emotional or physical).
2	The regional wall motion abnormalities usually extend beyond a single epicardial vascular distribution, and often result in circumferential dysfunction of the ventricular segments involved.
3	The absence of culprit atherosclerotic coronary artery disease, including acute plaque rupture, thrombus formation, and coronary dissection or other pathological conditions to explain the pattern of temporary LV dysfunction observed (e.g., hypertrophic cardiomyopathy, viral myocarditis).
4	New and reversible EKG abnormalities (ST-segment elevation, ST-segment depression, LBBB, T-wave inversion, and/or QTc prolongation) during the acute phase (3 months).
5	Significantly elevated serum natriuretic peptide (BNP or NT-proBNP) during the acute phase.
6	Positive but relatively small elevation in cardiac troponin measured with conventional assay (i.e., disparity between the troponin level and the amount of dysfunctional myocardium present).
7	Recovery of ventricular systolic function on cardiac imaging at follow-up (3 to 6 months).

**Table 3:** International Takotsubo Diagnostic Criteria All criteria should be met for TCM diagnosis [8]. TCM: Takotsubo cardiomyopathy, BNP: Brain natriuretic peptide, EKG: Electrocardiography

	International Takotsubo Diagnostic Criteria (requires all eight criteria)
1	Patients show transient left ventricular dysfunction (hypokinesia, akinesia, or dyskinesia) presenting as apical ballooning or midventricular, basal, or focal wall motion abnormalities. Right ventricular involvement can be present. Besides these regional wall motion patterns, transitions between all types can exist. The regional wall motion abnormality usually extends beyond a single epicardial vascular distribution; however, rare cases can exist where the regional wall motion abnormality is present in the subtended myocardial territory of a single coronary artery (focal TTS).
2	An emotional, physical, or combined trigger can precede the takotsubo syndrome event, but this is not obligatory.
3	Neurologic disorders (e.g., subarachnoid hemorrhage, stroke/transient ischaemic attack, or seizures) as well as pheochromocytoma may serve as triggers for takotsubo syndrome.
4	New EKG abnormalities are present (ST-segment elevation, ST-segment depression, T-wave inversion, and QTc prolongation); however, rare cases exist without any EKG changes.
5	Levels of cardiac biomarkers (troponin and creatine kinase) are moderately elevated in most cases; significant elevation of BNP is common.
6	Significant coronary artery disease is not a contradiction in takotsubo syndrome.
7	Patients have no evidence of infectious myocarditis.
8	Postmenopausal women are predominantly affected.
a	Wall motion abnormalities may remain for a prolonged period of time or documentation of recovery may not be possible. For example, death before evidence of recovery is captured.
b	Cardiac magnetic resonance imaging is recommended to exclude infectious myocarditis and diagnosis confirmation of takotsubo syndrome.

On an EKG, patients usually show ST-elevation, T-wave inversion, and left bundle-branch block [1]. Imaging can be obtained by TTE, transesophageal echocardiography, cardiac MRI, or cardiac ventriculogram, which will show the characteristic regional hypokinesis. Cardiac catheterization usually demonstrates unremarkable coronary arteries [1]. Troponin, brain natriuretic peptide (BNP), and N-terminal pro-BNP will be increased [1,11].

Takotsubo cardiomyopathy is unique among cardiomyopathies as it is mostly transient, and the affected heart has characteristic hyperkinetic basal segments with anteroseptal-apical dyskinetic ballooning of the left ventricle. It has four anatomic variants: apical ballooning, basal ballooning, midventricular ballooning, and focal ballooning of the left ventricle [12]. Interestingly, TCM incidence has a great gender and age imbalance. Templin et al. found that 89.8% of TCM cases were women, of which 79.1% were postmenopausal women in Western countries, even though there is male dominance in Japan [13]. The mechanism of this discrepancy may involve postmenopausal endothelial dysfunction and estrogen deficiency, leading to a decrease in cardiac protection [14,15].

The pathophysiology of TCM remains uncertain, but most evidence suggests that the sympathetic nervous system and catecholamines induced by emotional and physical stress may play an important role. Approximately two-thirds of TCM patients experience an increase in circulating catecholamines, which can lead to direct catecholamine toxicity [13]. Paur et al. demonstrated in a rat model that high intravenous epinephrine, but not norepinephrine, can reproduce apical myocardial depression like that seen in TCM [16]. This corresponds with other studies that show the cardiac apex has a higher concentration of beta-adrenergic receptors than other parts of the heart [16]. At high concentrations, epinephrine can act as a negative inotrope by binding to the β2-adrenergic receptor and temporarily switching it from an activating to an inhibiting role, which may explain the acute hypokinesis of the cardiac apex and why it is rapidly reversible [16]. Other possible pathogenic mechanisms include acute neurocardiogenic stunning of the heart [17], microvascular dysfunction at the apex of the heart [18], a decrease in glycolysis pathway metabolites leading to myocardial metabolite stunning [19], and severe heart inflammation [20].

Surgeries, which are physical stressors, can unsurprisingly induce TCM. In fact, Templin et al. reported that surgical stressors are the second most common physical stressors to induce TCM [13]. Case reports have demonstrated that TCM can occur in all types of surgery, from minor and minimally invasive procedures such as cataract extraction [21] and pregnancy termination [22], to more invasive surgeries such as mitral valve replacements [23] and carotid endarterectomies [24]. However, there are currently only two systematic reviews and no prospective trials on postoperative TCM, so the incidence of postoperative TCM and the correlation between surgical stress and TCM are unknown. Laparoscopic surgery is known to cause a reduction in cardiac output and an increase in systemic vascular resistance (SVR), which has been shown to persist after deflation of the abdomen [25-27]. This increase in SVR is most likely mediated by increased catecholamine release and may lead to postoperative TCM in susceptible patients. However, it is unknown if laparoscopic abdomen surgery is more likely to cause TCM than open abdomen surgery.

In the acute setting, TCM can lead to complications such as acute heart failure, cardiogenic shock with left ventricular outflow tract obstruction and non-obstruction, thrombosis, and even death. In the long run, it can recur. A cohort study conducted by Santoro et al. involving 1007 patients from the German and Italian Stress Cardiomyopathy Registry (GISCR) and 946 patients from the Spanish Registry for Takotsubo Cardiomyopathy demonstrated that the overall rate of in-hospital complications was 23.3% [28]. Furthermore, using the GISCR, Santoro et al. also found that 2.2% of TCM patients developed left ventricular thrombi [29]. However, the reported recurrence rate ranged from 1.8% to 4% [13,30]. Another study by Tenplin et al. reported that TCM patients had a death rate of 5.6% per patient year and a stroke or transient ischemic attack rate of 1.7% per patient year [13]. Similarly, Vallabhajosyula et al. showed that TCM had higher rates of stroke and heart failure than acute myocardial infarction [31].

Supportive treatment is usually sufficient for TCM as it is mostly transient. Left ventricle function typically returns spontaneously within 21 days of onset [32]. However, randomized controlled trials for TCM treatment are currently unavailable. If a patient presents with acute decompensating heart failure in shock, mechanical support may be preferred due to TCM's catecholamine toxicity etiology. However, low-dose epinephrine, as in the management of our patient, may be beneficial. There are currently no randomized trials to determine whether catecholamine or non-catecholamine treatment is better for TCM management. Low-dose levosimendan, dopamine, and dobutamine can also be used for hypotensive patients without substantial left ventricular outflow tract (LVOT) obstruction [33,34]. However, it is recommended to avoid dobutamine in patients with severe systolic dysfunction or LVOT obstruction with hypotension and shock [34]. When mechanical support is available, left ventricle assist devices, intra-aortic balloon pump support, and extracorporeal membrane oxygenation can be considered [35,36]. In stable patients, diuretics and vasodilators can be used for pulmonary congestion [32].

Due to the moderate risk of left ventricular thrombi development, patients should receive three months of dual antiplatelet therapy [29]. However, this must be balanced against the risk of bleeding after surgery. Short- and long-term use of angiotensin-converting enzyme inhibitors, angiotensin II receptor blockers, and/or beta-blockers may be beneficial. Nevertheless, there are mixed results in different studies regarding whether these medications can reduce mortality or recurrence [37,38].

As stress-induced catecholamines and psychiatric illness may play a significant role in TCM development, studies are being conducted to determine whether behavioral health therapy can reduce their recurrence. The prospective trial, Physical Exercise and Mental Wellbeing Rehabilitation for Acute Stress-induced Takotsubo Cardiomyopathy (PLEASE) study, will help determine if cognitive behavioral therapy and physical exercise can decrease TCM recurrence [39].

## Conclusions

In this report, we present the first case of postoperative TCM after an uncomplicated paraesophageal hernia repair. Takotsubo cardiomyopathy can mimic ACS and myocardial ischemia, making early diagnosis challenging. However, it should be considered a potential diagnosis for unexplained hypotensive postoperative patients, especially among postmenopausal women. Further research is needed to study the incidence, pathogenesis, and management of postoperative TCM.
